# Core-shell structure of LiMn_2_O_4_ cathode material reduces phase transition and Mn dissolution in Li-ion batteries

**DOI:** 10.1038/s42004-022-00670-y

**Published:** 2022-04-19

**Authors:** Chanikarn Tomon, Sangchai Sarawutanukul, Nutthaphon Phattharasupakun, Salatan Duangdangchote, Praeploy Chomkhuntod, Nattanon Joraleechanchai, Panyawee Bunyanidhi, Montree Sawangphruk

**Affiliations:** Center of Excellence for Energy Storage Technology (CEST), Department of Chemical and Biomolecular Engineering, School of Energy Science and Engineering, Vidyasirimedhi Institute of Science and Technology, 555 Moo 1 Payupnai, Wangchan District, Rayong, 21210 Thailand

**Keywords:** Batteries, Batteries

## Abstract

Although the LiMn_2_O_4_ cathode can provide high nominal cell voltage, high thermal stability, low toxicity, and good safety in Li-ion batteries, it still suffers from capacity fading caused by the combination of structural transformation and transition metal dissolution. Herein, a carbon-coated LiMn_2_O_4_ cathode with core@shell structure (LMO@C) was therefore produced using a mechanofusion method. The LMO@C exhibits higher cycling stability as compared to the pristine LiMn_2_O_4_ (P-LMO) due to its high conductivity reducing impedance growth and phase transition. The carbon shell can reduce direct contact between the electrolyte and the cathode reducing side reactions and Mn dissolution. Thus, the cylindrical cell of LMO@C//graphite provides higher capacity retention after 900 cycles at 1 C. The amount of dissoluted Mn for the LMO@C is almost 2 times lower than that of the P-LMO after 200 cycles. Moreover, the LMO@C shows smaller change in lattice parameter or phase transition than P-LMO, indicating to the suppression of λ-MnO_2_ phase from the mixed phase of Li_1-δ_Mn_2_O_4_ + λ-MnO_2_ when Li-delithiation at highly charged state leading to an improved cycling reversibility. This work provides both fundamental understanding and manufacturing scale demonstration for practical 18650 Li-ion batteries.

## Abstract (web summary)

Its high nominal voltage, thermal stability, and low toxicity render LiMn_2_O_4_ a highly promising cathode material for lithium ion batteries, but capacity fading due to unwanted side reactions during cycling remains an issue. Here, the authors show that carbon-coating a LiMn_2_O_4_ cathode reduces side reactions such as manganese dissolution and manganese oxide formation, thereby improving battery cycling stability.

## Introduction

Nowadays, rechargeable Li-ion batteries (LIBs) have become essential energy storage devices due to their high energy densities suitable for many electronic devices such as mobile phones and electric vehicles (EVs). The performance of LIBs is mainly determined by the cathode materials, which share ca. 30% of the total cost of LIB materials^1^. Thus, many extensive publications^2–5^ have been studied to develop effective cathode materials trying to increase energy and power densities and improve its stability, which are the important factors for the industrial production scale of LIBs. The face-centered cubic (fcc) spinel of LiMn_2_O_4_ (LMO) has received widespread attention due to its three-dimensional (3D) structure^6^, which can improve ion transport and power capability especially at high rates^7^. Moreover, LMO provides higher nominal voltage (*ca*. 4.0 V)^8^ than LiCoO_2_ (*ca*. 3.6 V)^9^, high thermal stability, low toxicity, and good safety which can simply be applied for large-scale energy storage applications^10,11^. However, the LMO is suffered from capacity fading caused by the combination of structural transformation and transition metal dissolution^12^. The Jahn–Teller distortion, occurred at the discharge state, introduces the structural change from cubic LiMn_2_O_4_ to tetragonal Li_2_Mn_2_O_4_ which causes the large anisotropic volume change (16%) as well as the structural damage^13,14^. To suppress the Jahn–Teller effect, Xiaohui Zhu et al^15^. presented a unique heterostructure design consisting of layered and spinel domains, disrupting the long-range Jahn–Teller ordering. As a result, they can enhance structural and electrochemical cycling stability.

Apart from Jahn–Teller effect, the Mn dissolution is one of the most important issues. The Mn dissolution from the spinel LiMn_2_O_4_ occurs in various situations such as at the aqueous acid solution, the high charging state, HF attack, and the over discharge state^16^. The main dissolution mechanism comes from the disproportionation reaction of Mn^3+^ to Mn^4+^ and Mn^2+^ as shown in [Eq. 1].

1 $${{{{{{\rm{2Mn}}}}}}}^{3+}\to {{{{{{\rm{Mn}}}}}}}^{4+}+{{{{{{\rm{Mn}}}}}}}^{2+}$$

In the overcharging process with Mn^4+^-rich state, the increasing Mn dissolution was caused by the instability of the delithiated structure at the end of the charging leading to loss of MnO to form a more stable single-phase structure. The phase transformation can cause the Mn dissolution from the spinel at the high potential as simply described in [Eq. 2]^16,17^.

2 $${{{{{{\rm{Li}}}}}}}_{{{{{{\rm{x}}}}}}}{{{{{{\rm{Mn}}}}}}}_{2}{{{{{{\rm{O}}}}}}}_{4}\to {{{{{{\rm{Li}}}}}}}_{{{{{{\rm{x}}}}}}}{{{{{{\rm{Mn}}}}}}}_{2-{{{{{\rm{y}}}}}}}{{{{{{\rm{O}}}}}}}_{4-{{{{{\rm{y}}}}}}}+{{{{{{\rm{yMnO}}}}}}}_{2}({{{{{\rm{soluble}}}}}})$$

After the Mn dissolution, the MnO (Mn^2+^) then dissolves into the electrolyte and migrates to the anode driven by the concentration gradient and/or electric field force and thereby deposits on the graphite surface so-called DMD process described in the [Eq. 3]^18^;

3 $${{{{{{\rm{yMn}}}}}}}^{2+}+{{{{{{\rm{Li}}}}}}}_{{{{{{\rm{x}}}}}}}{{{{{{\rm{C}}}}}}}_{6}\to {{{{{{\rm{yMn}}}}}}}^{0}({{{{{\rm{on}}}}}}\,{{{{{\rm{C}}}}}})+{{{{{{\rm{Li}}}}}}}_{{{{{{\rm{x}}}}}}-{{{{{\rm{2y}}}}}}}{{{{{{\rm{C}}}}}}}_{6}+{{{{{{\rm{2yLi}}}}}}}^{+}$$

The Mn^0^ gradually covers the anode surface, interfering with the lithium intercalation into the graphite and also increases the anode impedance^16^. Moreover, Delacourt at al^19^. proposed that the deposited Mn can react with carbonate and Li^+^ in the electrolyte forming the inactive LiCO_3_. This process consumes active Li ions over extended cycles, thus leading to severe capacity fading^12,20^.

The occurrence of Mn dissolution not only from the disproportionation reaction but also from HF attack from LiPF_6_ decomposition reacts with trace amount of water^21^. In addition, the capacity fading of the LMO cathode is deteriorated from the contraction/expansion of the crystal lattice during phase transition and also the generation of λ-MnO_2_ phase during charging process^22^.

Various strategies have been studied to develop the LMO material such as the synthesis of nanomaterials^23,24^, ion doping^25–27^, and surface coating^28–30^. Nonetheless, the doping and synthesis of nanomaterials lead to the reduction of overall cell capacity due to the electrochemically inactive cation dopants^31,32^ and the poor packing density of nano-grains^33,34^, respectively. Moreover, they cannot prevent the Mn dissolution from side reactions occurring at the interface between the electrode and the electrolyte during the charge/discharge processes. On the other hand, the surface coating can minimize the direct contact between the active cathode and the electrolyte, reduce Mn dissolution, and alleviate the Mn deposition on the anode surface resulting in less impedance growth^35–37^. However, the surface engineering/coating normally requires complex synthesis processes and high cost, for example, wet chemical coating and atomic layer deposition which will be ineffective for uses in commercial-scale LIBs.

Recently, a surface coating as the core-shell morphology using a simple and solvent-free mechanofusion process has been proposed as an efficient method for the development of cathode materials for scalable LIBs^37,38^. Specifically, carbon coating has received much attention because carbon can facilitate the continuous electron pathway through the coated shell leading to an improved active material utilization^39,40^. Moreover, some previous publications show that the carbon coating can improve the stability and reduce the Mn dissolution^40^. Nevertheless, the fundamental understanding on the minimized phase transformation from the carbon-coated LMO core-shell has not yet been investigated. In this work, the mechano-thermal carbon-coated LMO core-shell can suppress the phase transformation, especially reducing the occurrence of an unstable two-phase region (Li_1-δ_Mn_2_O_4_ + λ-MnO_2_ mixed phase) at high potential.

Herein, the nano-sized conductive carbon coated on the LMO (LMO@C) cathode was synthesized using a dry mechanofusion technique. After the coating process, the 18650 cylindrical LIBs of LMO@C cathode coupled with graphite anode were fabricated in a dry room. The LMO@C exhibits superior cycling stability compared with pristine-LMO (P-LMO), which can mainly attribute to the reduction of Mn dissolution-migration-deposition (DMD) process as investigated by the post-mortem analyses using ex situ SEM/EDS, ICP-OES, and ex situ XPS techniques. In addition, the structural transformation is explored by *in operando* X-ray diffraction (XRD) during charging and discharging. The in situ XRD of P-LMO reveals the rapid drop in lattice parameter (a/Å) when Li deintercalated over *ca*. 70%, indicates to the generation of λ-MnO_2_ phase mixed with Li_1-δ_Mn_2_O_4_^22^. However, the LMO@C exhibits lower change in lattice parameter implying to the lower generation of λ-MnO_2_ in Li_1-δ_Mn_2_O_4_ + λ-MnO_2_ mixed phase. These results confirm that the carbon shell can minimize Mn dissolution and stabilize phase transformation, which will be beneficial for improving the battery lifetime.

## Results and discussion

### Physicochemical properties

**Fig. 1:**
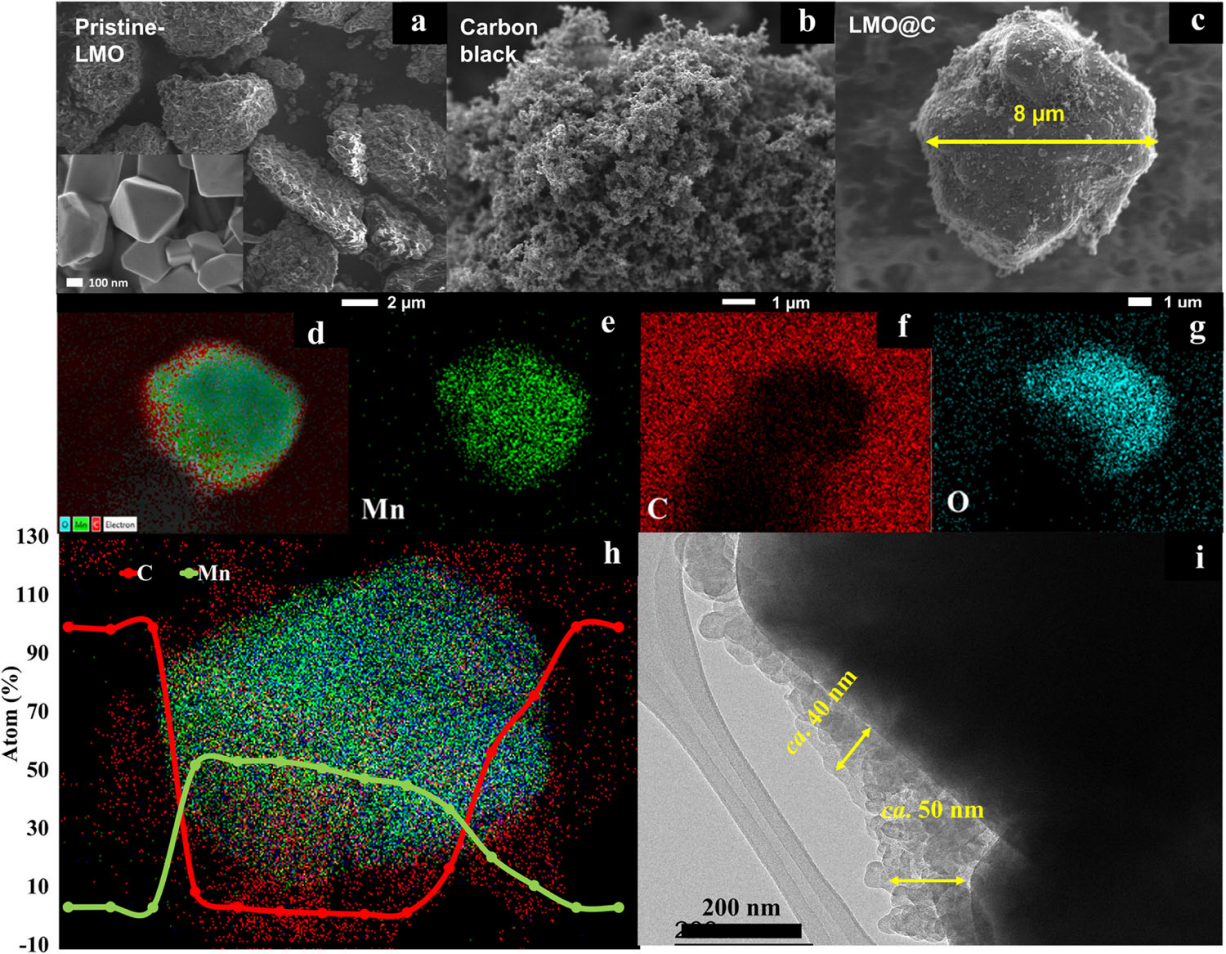
Morphology of pristine-LMO and LMO@C materials. SEM images of (**a**) pristine-LMO, (**b**) carbon black, and (**c**) LMO@C. **d**–**g** SEM-EDS of LMO@C. **h** STEM-EDS line scanning of LMO@C. **i** HR-TEM images of LMO@C.

The morphologies of pristine-LMO (P-LMO), carbon black, and LMO@carbon core@shell (LMO@C) materials synthesized by the mechanofusion process were investigated by FE-SEM/EDS, HRTEM, and STEM-EDS as shown in Fig. 1. From Fig. 1a, the P-LMO shows the secondary particle consisting of primary particles with the octahedral structure. Figure 1b shows the morphology of carbon nanospheres with individual particle size of *ca*. 50 nm. After the mechanofusion of P-LMO with carbon nanoparticles, the applied high compression and shear forces can fuse the carbon nanoparticles into the surface of LMO providing the core-shell like structure with the diameter of *ca.* 8 µm (Fig. 1c)^37,41^. EDX and STEM-EDS results (Fig. 1d–h) confirm the uniform carbon coating in which the atomic ratio of C at the shell decreases to 0% when approaching to the center of LMO@C particle. In contrast, the percentage of Mn atom increased when approaching to the center of the particle. The HRTEM image (Fig. 1i) shows the carbon shell thickness of *ca*. 40–50 nm composing of 2–4 overlapping carbon nanospheres with no void spaces at the interface between the core particles and carbon shells. The effective adherence of carbon and LMO can be attributed to the strong compressive force and high heat energy generated during the mechano-thermal process^42,43^. The carbon coating layer is expected to improve electrical conductivity of the material and suppress the Mn dissolution as it can prevent the direct contact from the electrolyte and prohibit the acute HF attack. Note, HF is generated from the decomposition of LiPF_6_ as shown in [Eq. 4–6]^21^.

4 $${{{{{{\rm{LiPF}}}}}}}_{6}({{{{{\rm{s}}}}}})\leftrightarrow {{{{{\rm{LiF}}}}}}({{{{{\rm{s}}}}}})+{{{{{{\rm{PF}}}}}}}_{5}({{{{{\rm{g}}}}}})$$

5 $${{{{{{\rm{H}}}}}}}_{2}{{{{{\rm{O}}}}}}({{{{{\rm{l}}}}}})+{{{{{{\rm{PF}}}}}}}_{5}({{{{{\rm{g}}}}}})\to {{{{{\rm{2HF}}}}}}({{{{{\rm{l}}}}}})+{{{{{{\rm{POF}}}}}}}_{3}({{{{{\rm{g}}}}}})$$

6 $${{{{{{\rm{2LiMn}}}}}}}_{2}{{{{{{\rm{O}}}}}}}_{4}({{{{{\rm{s}}}}}})+{{{{{{\rm{4H}}}}}}}^{+}({{{{{\rm{l}}}}}})\to {{{{{{\rm{3MnO}}}}}}}_{2}({{{{{\rm{s}}}}}})+{{{{{{\rm{Mn}}}}}}}^{2+}({{{{{\rm{l}}}}}})+{{{{{{\rm{2Li}}}}}}}^{+}+{{{{{{\rm{2H}}}}}}}_{2}{{{{{\rm{O}}}}}}({{{{{\rm{l}}}}}})$$

Moreover, the Mn dissolution can occur from the instability of the delithiated structure at high SOC leading to the loss of MnO and forming λ-MnO_2_ as shown in [Eq. 1]^44^.

The structure of P-LMO and LMO@C were investigated using XRD as shown in Fig. S1a. The XRD patterns of P-LMO and LMO@C can be well indexed to the cubic spinel phase of *Fd(-)3* *m* space group (JCPDS 98-005-0256), corresponding to (111), (311), (222), (400), (331), (511), (440), and (531) planes. In this structure, lithium, manganese, and oxygen reside on the 8a (tetrahedral sites), 16d (octahedral sites), and 32e Wyckoff sites, respectively^45^. No obvious peaks from carbon coating could be observed by XRD, due to its small quantity as well as its amorphous characteristic^40,46^. However, in the inset image of Fig. S1a, the slight peak shift toward higher 2Theta corresponded to the compression effect during the mechanofusion process^47,48^. To gather detailed information of the structure, Rietveld refinement was performed using the Rietica software. The refinement data are presented in Fig. S2 and Table S1, respectively. The similar lattice parameters of 8.233 Å in both P-LMO and LMO@C indicated that the carbon coating does not alter the unit cell structure of the active material.

Figure S1b shows the Raman spectra of P-LMO and LMO@C. Normally, LMO with *Fd(-)3* *m* space group has five Raman-active modes represented by the species of A_1g_ **+** E_g_ **+** 3F_2g_^49,50^. Both of P-LMO and LMO@C present a dominant peak at 630 cm^−1^ relating to Mn-O vibrational mode in the MnO_6_ octahedral. Moreover, two bands located at *ca*. 492 and 585 cm^−1^ refer to high-frequency scattering bands of F_2g_(2) and F_2g_(3) that result from the large and small Mn atom vibrations, respectively^49,51,52^. The E_g_ band located at 382 cm^−1^ also corresponds to the O vibration^53^. It can be noticed that there is no obvious peak shift after the mechanofusion of carbon coating process. Thus, the mechanofusion process did not change the characteristics of LMO. However, the two distinct peaks in LMO@C were founded at 1360 and 1592 cm^−1^, corresponding to the A_1g_ and E_2g_ vibrational modes of the disorder (D) and graphitic (G) bands, which are the typical peaks of carbon. A broad peak at 2701 cm^−1^ indicates the 2D band of carbon^40,54^. The appearance of I_D_, I_G_, and 2D bands only in LMO@C indicates to the carbon content from the carbon coated on LMO@C.

The TGA of P-LMO and LMO@C was used to investigate the amount of carbon shell coated on the LMO core as shown in Fig. S1c. The TGA profile of P-LMO shows a slightly weight loss with only 1.58 wt.% during the whole temperature measurement. Conversely, the LMO@C exhibits the mass loss from residual moisture and other volatiles with *ca*. 2.4 wt.% before 400 °C, and then rapidly drop of *ca*. 10 wt.% at 400 °C according to the oxidation of carbon at the shell^37^.

**Fig. 2:**
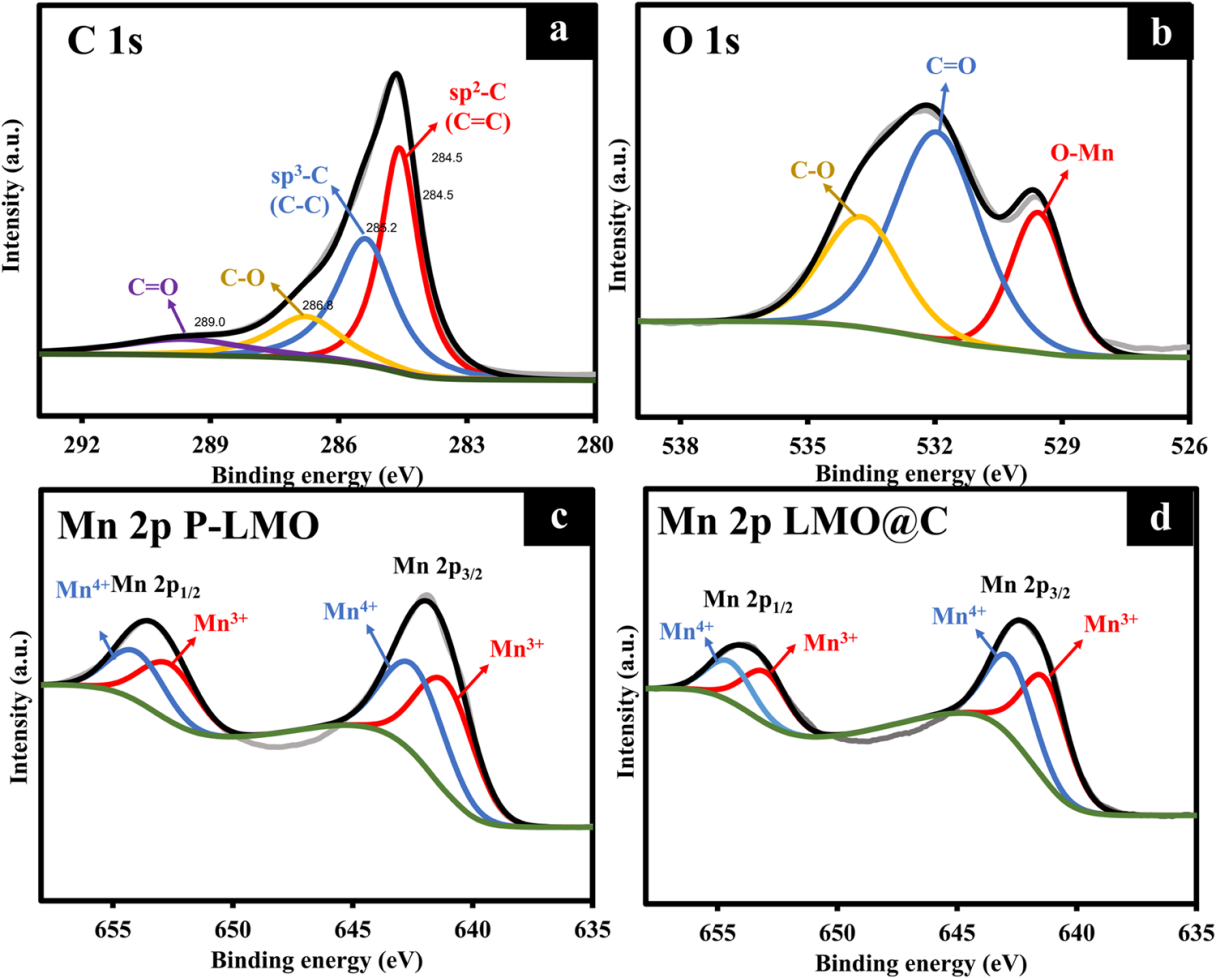
The elemental composition and the oxidation state of P-LMO and LMO@C. **a**–**d** Narrow-scan XPS spectra of (**a**) C 1 s of LMO@C, (**b**) O 1 s of LMO@C, (**c**) Mn 2p of P-LMO, and (**d**) Mn 2p of LMO@C.

The elemental composition and the oxidation state of P-LMO and LMO@C were further characterized by the XPS technique as shown in Fig. S1d and Fig. 2. In wide-scan XPS spectra, the sharp peaks of C 1 s, O 1 s, and Mn 2p are located at *ca*. 245, 532, and 642 as well as 654 eV, respectively. The C 1 s intensity in LMO@C is significantly higher than P-LMO due to the existence of carbon shell. The peak deconvolution and fitting of narrow scans (Fig. 2) were carried out using Gaussian−Lorentzian shaped peaks based on the Shirley background correction. The C1s spectrum of LMO@C (Fig. 2a) displays four deconvoluted peaks comprising of sp^2^-C, sp^3^-C, C–O, and C**=**O located at 284.5, 285.2, 286.8, and 289.0 eV, respectively^55,56^. The O 1 s spectrum of LMO@C (Fig. 2b) shows three main components at 529.3, 531.8, and 533.4 eV correlating to O^2-^ anions of LiMn_2_O_4_^57^, C**=**O, and C–O–C (surface adsorbed species), respectively^55,58,59^. Two typical peaks corresponding to a spin–orbit splitting of Mn 2p_3/2_ and 2p_1/2_ are observed in the P-LMO (Fig. 2c) and LMO@C spectra (Fig. 2d)^60^. Both of them present similar splitting binding energy about 11.6 eV agreeable well with the previous reports^40,61^. The deconvoluted Mn 2p of P-LMO displays the mixing of Mn^3+^ and Mn^4+^ for which the binding energies of Mn^3+^ are 641.2 eV (2p_3/2_) and 652.7 eV (2p_1/2_), whereas those of Mn^4+^ are 642.6 eV (2p_3/2_) and 654.2 eV (2p_1/2_). For the LMO@C, the binding energies of Mn^3+^ are 641.3 eV (2p_3/2_) and 652.9 eV (2p_1/2_) and those of Mn^4+^ are 642.7 eV (2p_3/2_) and 654.5 eV (2p_1/2_). The ratios of Mn^3+^/Mn^4+^ of P-LMO and LMO@C are 1.17: 1 and 1.07:1, respectively. The Mn^3+^/Mn^4+^ ratio of LMO@C is lower than that of P-LMO and also close to an expected theoretical ratio of 1:1. In principle, the electrochemically active Mn^3+^ is not stable so the higher ratio in the structure can lead to the Mn^3+^ disproportination^40,55^. According to these results, the carbon coating may be able to reduce the Mn dissolution.

### Electrochemical property of coin-cell batteries

**Fig. 3:**
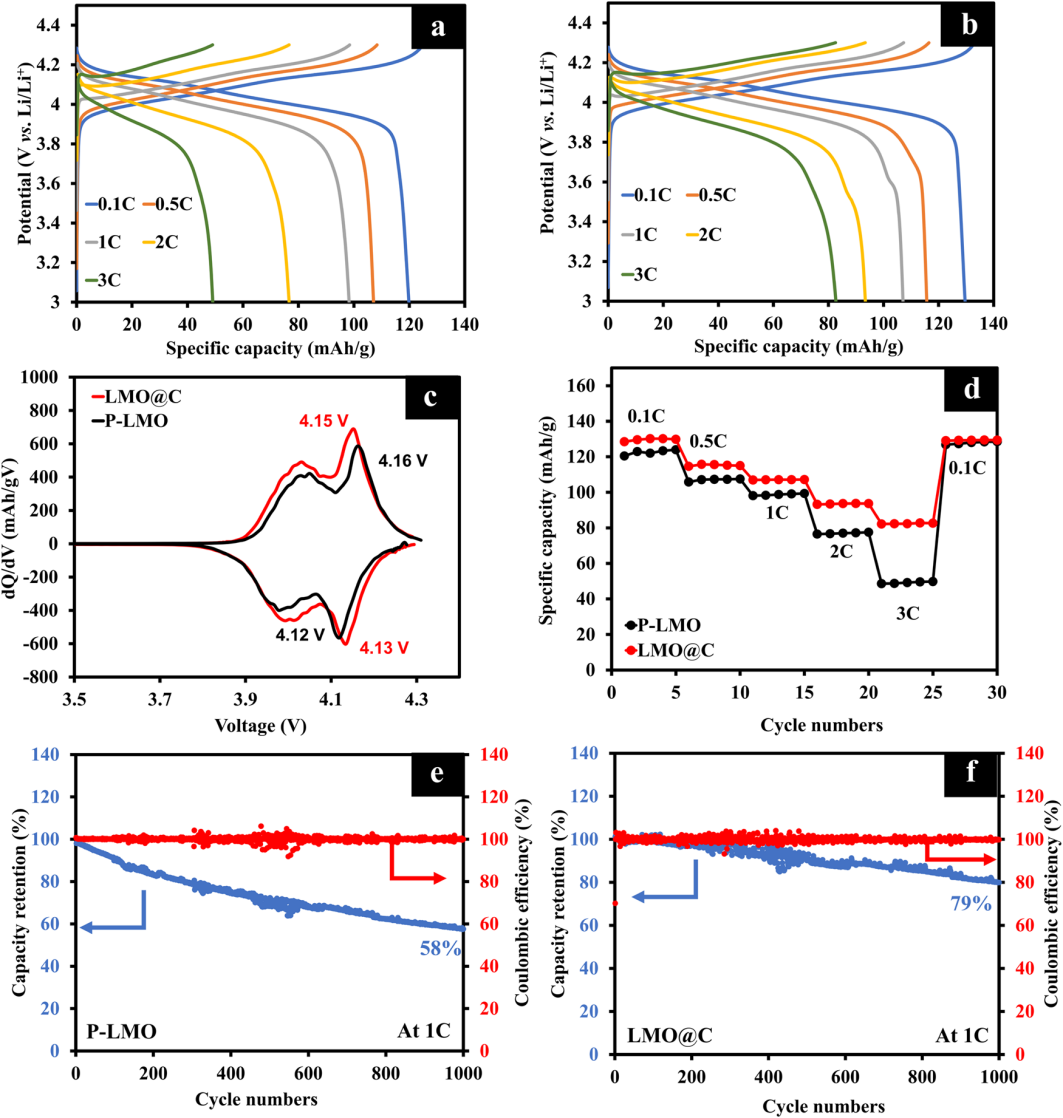
Electrochemical performance of half-cell configurations for Li//P-LMO and Li//LMO@C. **a**–**b** Galvanostatic charge/discharge curves at different C-rates of (**a**) P-LMO and (**b**) LMO@C. **c** The dQ/dV curves of P-LMO and LMO@C. **d** The rate capability of P-LMO and LMO@C. **e**–**f** The cycling stability test of (**e**) P-LMO, and (**f**) LMO@C at 1C.

The electrochemical tests were firstly performed in the half-cell configurations of Li//P-LMO and Li//LMO@C. These results are shown in Fig. 3. According to charge/discharge profiles (Fig. 3a, b) over the voltage range of 3.0–4.3 V vs. Li/Li^+^, both P-LMO and LMO@C provide two distinct plateaus at around 4.0 and 4.15 V vs. Li/Li^+^. These corresponds to the two reversible deintercalation/intercalation reactions of Li^+^ from the tetrahedral sites in the dQ/dV results (Fig. 3c), described in Eqs. 7 and 8^62,63^.

7 $${\rm LiM}{\rm n}_{2}{\rm O}_{4}\,\leftrightarrow 0.5{\rm Li}^{+}+0.5{\rm e}^{-}+{\rm Li}_{0.5}{\rm Mn}_{2}{\rm O}_{4}$$

8 $${\rm Li}_{0.5}{\rm Mn}_{2}{\rm O}_{4}\leftrightarrow 0.5{\rm Li}^{+}+0.5{\rm e}^{-}+2\lambda -{\rm MnO}_{2}$$

During the charging process, half of the Li^+^ ions are de-intercalated from the tetrahedral sites in LiMn_2_O_4_ (Eq. 7) and associated with the first plateau at *ca*. 4.0 V vs. Li/Li^+^. After that, further de-lithiation continuously occurs to form the final product with λ-MnO_2_ (Eq. 8) that can be indexed to the second plateau at *ca*. 4.15 V vs. Li/Li^+^^64^.

According to Fig.3c, the peak potential difference (∆E_p_) between the oxidation and the reduction can suggest the polarization degree of the battery^65^. The lower ∆E_p_ values of LMO@C (20 mV) than P-LMO (40 mV) indicate that LMO@C has a smaller electrochemical reaction polarization than P-LMO. Moreover, the rate performance and profiles at different C-rates for P-LMO and LMO@C are shown in Fig. 3d. The discharge capacities of LMO@C are about 129.6, 115.7, 107.1, 93.7, and 82.3 mAh g^−1^ obtained from 0.1 C, 0.5 C, 1 C, 2 C, and 3 C, respectively. On the other hand, P-LMO exhibits the specific discharge capacities of 121.3, 107.3, 96.7, 84.1, and 66.3, and 48.0 mAh g^−1^ corresponding to 0.1 C, 0.5 C, 1 C, 2 C, and 3 C, respectively. The low specific capacities at high C-rate are attributed to the increasing electrode polarization, attributed from the increasing overpotential and the increasing internal resistance or IR drop^66^. As compared with the P-LMO, the LMO@C shows lower polarization and higher capacity at high C-rates which could be ascribed to the improved electron conductivity, thereby increasing the active material utilization^40^.

Furthermore, the long-term cycling test was carried out to confirm the improved electrochemical property of LMO@C compared with P-LMO at 1 C (Fig. 3e, f). Obviously, the LMO@C provides superior capacity retention of 79% after 1000 cycles which is considerably higher than that of the P-LMO (58% capacity retention). The coulombic efficiency is maintained around 99% for the 1000 cycles for both LMO@C and P-LMO. The enhanced cycling stability from the carbon coating could be explained by (i) less direct contact between the electrode and the electrolyte, (ii) suppression of Mn dissolution, migration into an electrolyte, and deposition at anode surface so-called “DMD process” which will be further investigated by ex situ SEM, ICP-OES, and XPS in the next sections, (iii) increasing of phase stabilization that will be examined by in situ XRD, and (iv) the improved conductivity^67–69^.

### Electrochemical properties of cylindrical 18650 cells

To demonstrate the practical application and scalable process of LMO@C, the 18650 cylindrical LIBs were fabricated in a dry room with dew point temperature of −40 °C for all production units except for the electrolyte injection unit at −55 °C. The P-LMO cylindrical LIBs were assembled for comparison. Both LMO@C and P-LMO were coupled with graphite anode and with a commercial LMO electrolyte (EC+DMC+DEC). Fig. S3 illustrates the diagrams and photographs of the 18650 LIBs manufacturing process. The four-point probe measurements (Table S2) were also carried out to measure the resistance of each electrode. Interestingly, LMO@C cathode provides a resistivity just only 0.47 mΩ/cm which is five times lower than P-LMO cathode. This indicates that the carbon shell can dramatically increase conductivity of the electrode^70–72^.

**Fig. 4:**
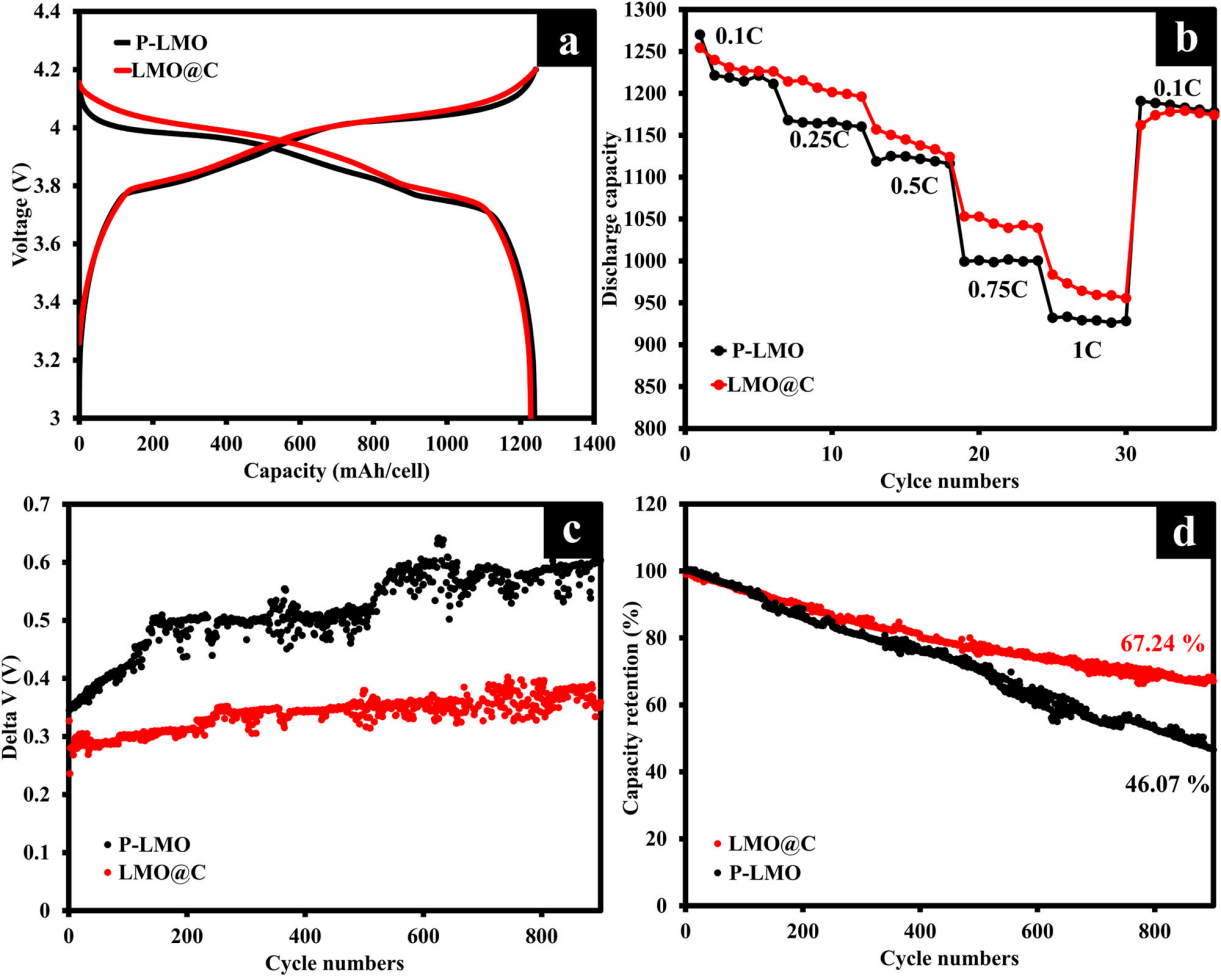
Electrochemical performances of cylindrical 18650 P-LMO//graphite and LMO@C//graphite cells. **a** Galvanostatic charge/discharge profiles of P-LMO and LMO@C cells. **b** Rate-capability of P-LMO and LMO@C cells. **c** Delta V vs. cycle of P-LMO and LMO@C cells. **d** Cycling stabilities of P-LMO and LMO@C cells.

To determine the electrochemical performances (Fig. 4), the LMO@C//graphite and P-LMO//graphite cells were tested in a voltage range of 3.0–4.2 V at 0.1 C (Fig. 4a) by using CC-CV charging method. The results show the same capacity of around 1277 mAh/cell in LMO@C as well as P-LMO with the nominal voltage of 3.93 V. Whilst, the rate-capability (Fig. 4b) of LMO@C//graphite is slightly better at the high rates of 0.75 C and 1 C which could be attributed to the enhanced conductivity from carbon coating as discussed in previous section.

Figure 4c shows the difference between average charge and discharge voltage (∆V) vs. cycle numbers for P-LMO//graphite and LMO@C//graphite cells. The ∆V values during cycling can describe the impedance growth and polarization in the cells^73,74^. The P-LMO//graphite cell exhibits significant increase in ∆V during cycling, indicating an impedance increase. In contrast, the LMO@C//graphite cell shows smaller ∆V due to the smaller polarization in the cell and lower impedance compared with P-LMO//graphite.

Figure S4a and S4b present the dQ/dV over 100 cycles at 0.1 C for P-LMO and LMO@C, respectively. Both provide two distinct oxidation-reduction peaks located at *ca*. 4.07/3.94 and 3.74/3.83 V, indicating to the reversible deintercalation/intercalation of Li^+^ from the tetrahedral sites as mentioned in Eqs. 7 and 8. The peak potential difference (∆E_p_) values of 1st and 100th cycles of P-LMO are 80.6 and 137 mV, respectively. Whilst the LMO@C shows lower peak potential different at 100th cycle (98 mV) compared to the 1st cycle (80.7 mV). This indicates that the LMO@C exhibits better reversibility than the P-LMO^75^.

The constant current stability test was carried out at a high rate of 1 C for 900 cycles as shown in Fig. 4d. The capacity retention of P-LMO//graphite cell dramatically decreased to 46% at 900 cycles, that could be attributed to the Mn dissolution, the Mn redeposition at anode site, mixed phase transition, and poor conductivity. In contrast, the LMO@C can maintain the capacity retention at 67.24% after 900 cycles, suggesting that carbon shell can improve the stability of cylindrical LIBs.

### Post-mortem analysis

The post-mortem analyses including ex situ SEM, ex situ XPS, ex situ ICP-OES, and in situ XRD were carried out to investigate the origin of capacity fading. In principle, the DMD process has been generally accepted that it is the crucial factor giving rise to the capacity fade of Mn-based LIBs^16^. Figure S5 presents the ex situ SEM images and EDS mapping of graphite anode disassembled from the P-LMO//graphite (Fig. S5a) and LMO@C//graphite (Fig. S5b) after 200 cycles. According to Fig. S5, the % Mn content on graphite from P-LMO (2%) is approximately two times higher than graphite from LMO@C (1.1%), indicating to the reduction of DMD process in LMO@C. Moreover, the results from the ICP-OES measurement reveal the amount of Mn deposition on the graphite surface after 200 cycles. According to Table S3, the concentration of Mn deposited on anode side of P-LMO//graphite, and LMO@C//graphite cells were 0.022 mg/l and 0.01 mg/l, respectively. These results agree well with ex situ SEM and EDS mapping.

**Fig. 5:**
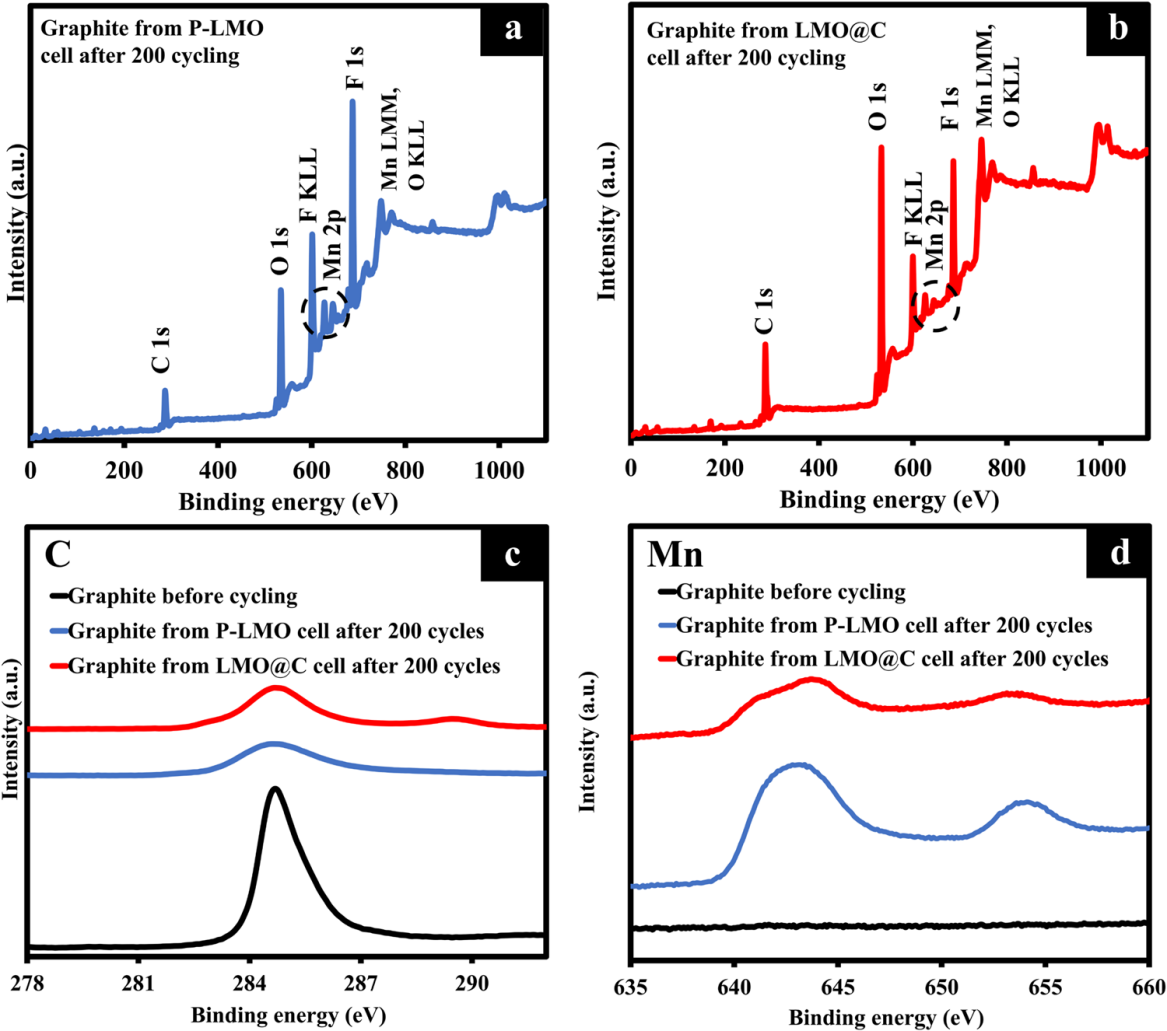
Ex situ XPS of cathodes disassembled from P-LMO//graphite and LMO@C//graphite cells after cycling. **a**–**b** Wide-scan XPS of (**a**) P-LMO electrode, and (**b**) LMO@C electrode. **c**–**d** Narrow-scan XPS of (**c**) C 1 s orbitals, and (**d**) Mn 2p orbital of controlled graphite (before cycling), disassembled graphite from P-LMO//graphite and LMO@C//graphite cells after 200 cycles.

The surface analysis by the ex situ XPS (Fig. 5 and Table S4) of graphite anodes after cycling were revealed as the strong evidence for the occurrence of DMD process. Figure 5a, b show the wide-scan spectra from graphite anodes disassembled from P-LMO//graphite and LMO@C//graphite, respectively. These two anodes show the main compositions of C, O, Mn, and F, in which F came from the decomposition of LiPF_6_ or SEI layer^76^. Furthermore, Fig. 5d displays the narrow scan Mn 2p spectra on graphite surfaces before and after cycling for which the cycled P-LMO//graphite and LMO@C//graphite disassembled for XPS measurement. After cycling, the Mn was detected in both anodes from the cycled P-LMO and LMO@C cells, but the Mn intensity from the cycled P-LMO cell is significantly higher than that of the cycled LMO@C cell. The Mn/C ratios of anodes from the cycled P-LMO and LMO@C are 0.25 and 0.17 (Table S4), respectively. Therefore, this clearly indicated that the carbon coating can suppress the Mn dissolution.

Note that the observed Mn peaks situated around 641 eV indicate manganese divalent or trivalent such as MnO and Mn_2_O_3_^77^. These results suggest the irreversible decomposition and thick deposition by Mn (II) dissolution^77^. After the Mn (II) dissolution [Eq.2], metallic Mn was first deposited on the anode surface and subsequently reacts with organic species in electrolyte as shown in [Eqs. 9 and 10]^18,19,78,79^.

9 $${{{{{{\rm{Mn}}}}}}}^{0}+{{{{{\rm{EC}}}}}}\to {{{{{{\rm{MnCO}}}}}}}_{3}+{{{{{{\rm{C}}}}}}}_{2}{{{{{{\rm{H}}}}}}}_{4}$$

A conversion reaction could occur between MnCO_3_ and Li^+^ as follows:

10 $${{{{{{\rm{MnCO}}}}}}}_{3}+{{{{{{\rm{2Li}}}}}}}^{+}+{{{{{{\rm{2e}}}}}}}^{-}\leftrightarrow {{{{{{\rm{Li}}}}}}}_{2}{{{{{{\rm{CO}}}}}}}_{3}+{{{{{{\rm{Mn}}}}}}}^{0}$$

The [Eq. 10] occurs reversibly during cycling and changes the morphology of the SEI continuously, resulting in the occurrence of cracks and pores in SEI layer^19^. Moreover, the Mn ions dissolute or strip from the cathode and redeposited or plated on the anode surface, which can block Li^+^ diffusion pathways leading to the increasing impedance^80^. These two reasons can directly affect the capacity fading of the practical cell.

### *In operando* XRD

**Fig. 6:**
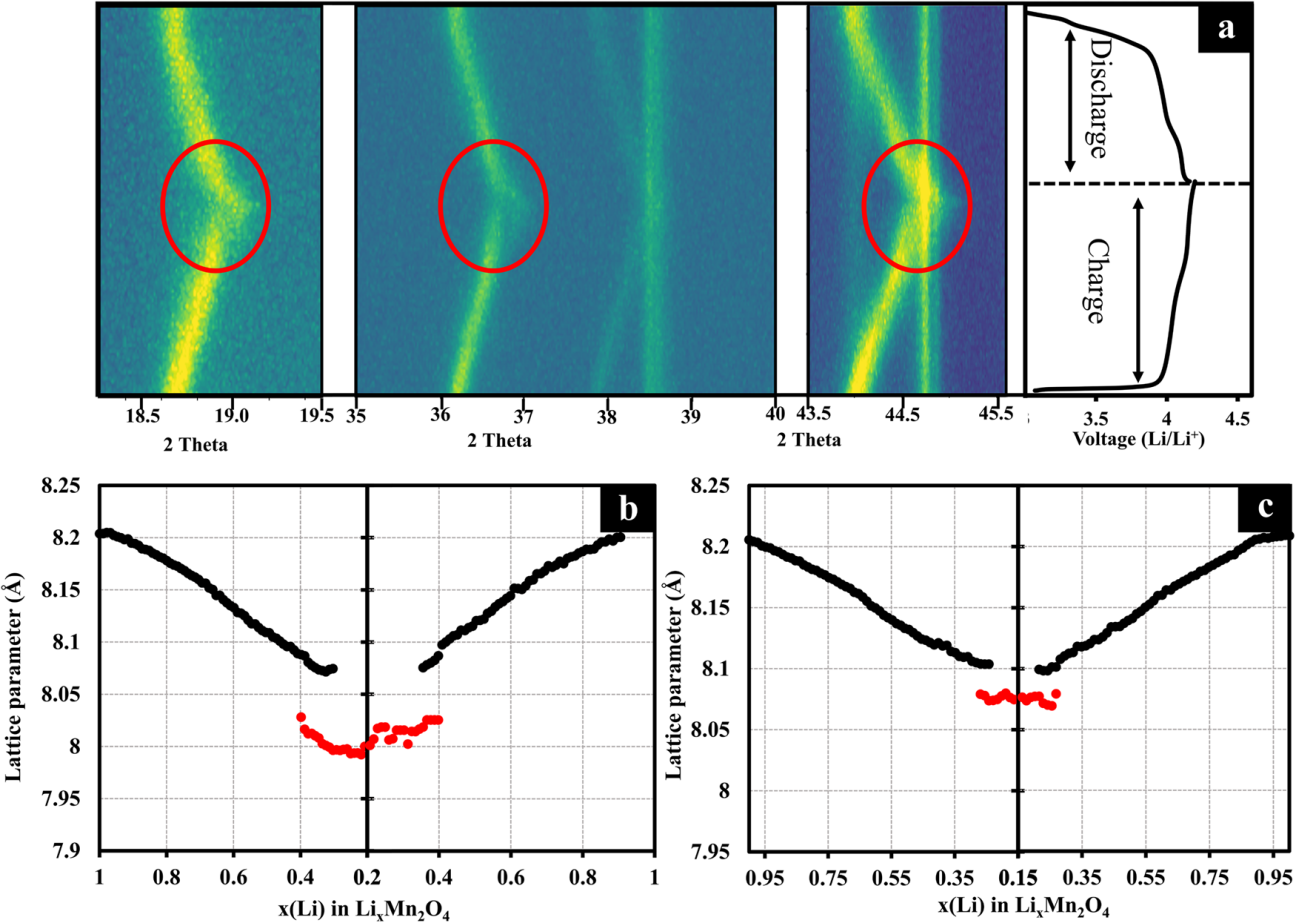
Structural transition altering in lattice parameters of P-LMO and LMO@C cathodes upon lithium deintercalation/ intercalation. **a** *In operando* XRD patterns during charging and discharging. **b**–**c** The correlation between lattice parameter and the amount of Li in LiMn_2_O_4_ in (**b**) P-LMO, and (**c**) LMO@C.

The structural transition of P-LMO and LMO@C cathodes upon lithium deintercalation/ intercalation was investigated by *in operando* XRD as shown in Fig. 6. *In operando* XRD of P-LMO (Fig. S6) and LMO@C (Fig. 6a) cathodes show two-phase transition stages during charge/discharge process relating to two peaks observed in its dQ/dV curves. In the first charging state with less than 50% Li extraction, the XRD peaks gradually shifted to higher 2Theta (Fig. S6 and Fig. 6a) according to the decrease in lattice parameters (Fig. 6b, c). The increasing in the average Mn oxidation state led to the decrease in ionic radius and cell dimensions^81–83^. In the following region, when Li extraction exceeds 50% (*ca*. 4.05 V), further de-intercalation of Li^+^ ion from the Li_0.5_Mn_2_O_4_ induces a solid-solution reaction to form Li-deficient Li_1-δ_Mn_2_O_4_^12,22^. Furthermore, at high state of charge (SOC), the mixed phase of Li_1-δ_Mn_2_O_4_ and λ-MnO_2_ was formed, which can be noticed by the occurring of new phase at the end of charge (red circle) in Fig.6a and Fig. S6. After Li re-intercalation (discharge process), the XRD peaks shifted back to lower 2Theta, indicating the existence of reversible phase transformation to cubic LiMn_2_O_4_^81^.

From previous reports^62,81,82,84^, there are three phases in the phase transition including a single-phase LiMn_2_O_4_, the mixed phases of LiMn_2_O_4_ + Li_1-δ_Mn_2_O_4_ and the mixed phases with λ-MnO_2_. Nevertheless, the phase between single-phase LiMn_2_O_4_ and the mixed phases of LiMn_2_O_4_ + Li_1-δ_Mn_2_O_4_ is difficult to be distinguished. In contrast, the mixed phase of λ-MnO_2_ can be observed from the appearance of λ-MnO_2_ lattice parameter at *ca*. 8.02–8.07 Å and the presence of new phase at high angle described in the circle in Figs. S6 and 6a^81^.

In P-LMO (Fig. 6b), the lattice parameter continuously decreased from 8.20 Å to 8.0 Å during charging process with the existence of λ-MnO_2_ phase at *x*Li^+^ of *ca*. 0.4–0.2 (~60% Li deintercalation). Whilst, the LMO@C (Fig. 6c) exhibits much smaller amounts of λ-MnO_2_ phase, presented after Li deintercalated over *ca*. 75% (*x*Li^+^ = *ca*. 0.25–0.15).The smaller appearance of λ-MnO_2_ phase and lower changing in lattice parameter of LMO@C indicating to the lower generation of λ-MnO_2_ and suppressed contraction/expansion of the crystal lattice^62^.

**Fig. 7:**
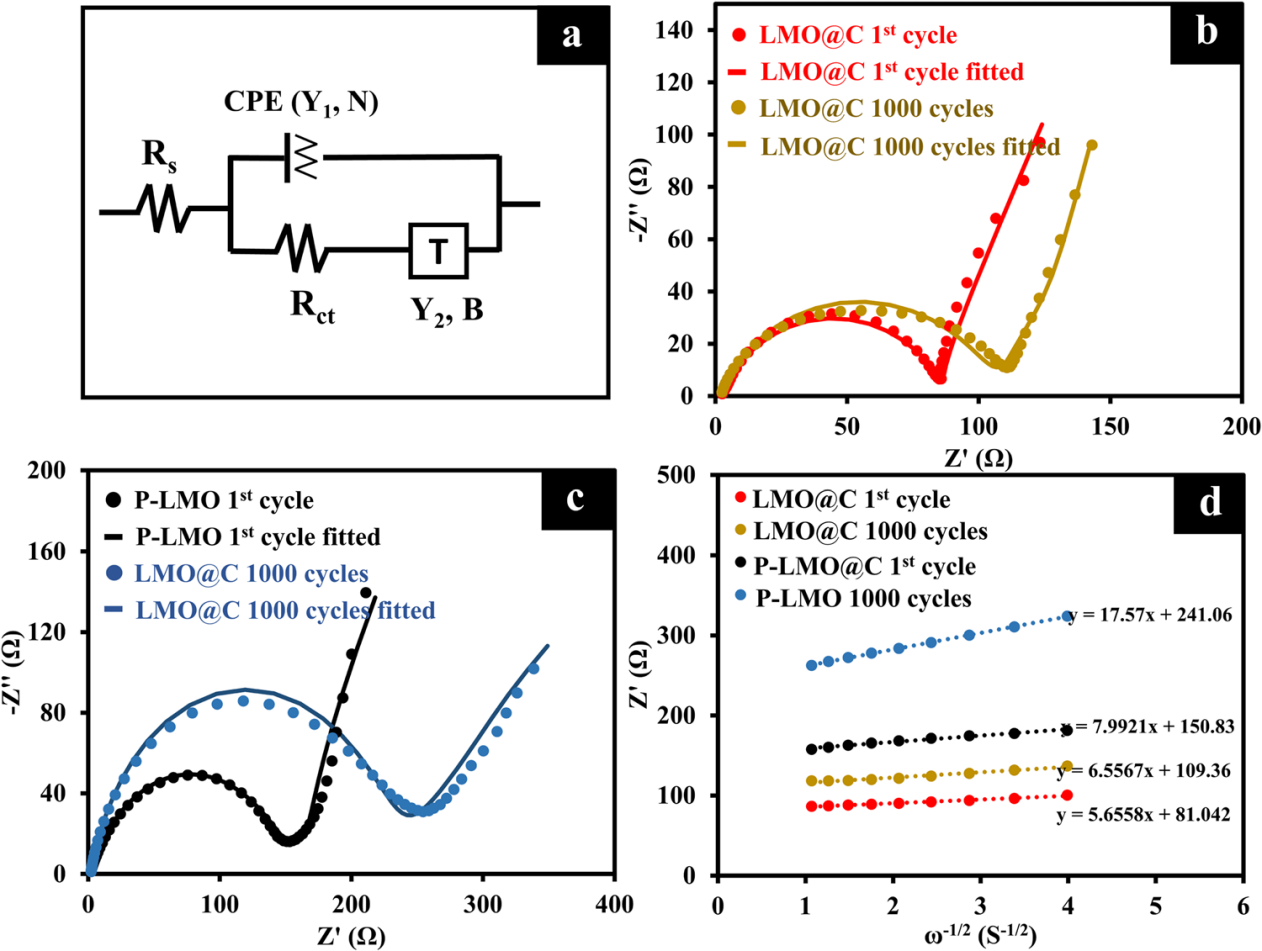
Impedance measurement of half-cell P-LMO and LMO@C representing the cathode degradation after cycling. **a** The fitted equivalent circuit. **b**–**c** EIS curves and fitted curves in the half-cell CR2032 of (**b**) LMO@C, and (**c**) P-LMO before and after cycling. **d** The plot between Z’ and ω-1/2 at low-frequency region for P-LMO and LMO@C.

To further clarify the cathode degradation, the impedance measurement with the half-cell configuration of P-LMO and LMO@C was used to consider the cathode properties and Li-ion diffusion coefficient as shown in Fig. 7. The Nyquist plots can be well-fitted with the equivalent circuit, as shown in the Fig. 7a. The T (Tangent hyperbolic) symbol corresponds to the finite space Warburg. According to Fig. 7 and Table S5, before cycling, the R_ct_ of LMO@C cell is significantly lower than that of P-LMO indicating the improvement of conductivity from carbon coated. Moreover, after cycling, the R_ct_ of P-LMO cell is rapidly increased for 86 Ω, while the R_ct_ of LMO@C is increased only 19.6 Ω. The four times lower in the impedance growth can be assumed from the decreasing Mn dissolution at the LMO@C cathode^85^. The encapsulated carbon shell can reduce the direct contact between the electrolyte and the cathode reducing side reactions and the Mn dissolution.

The Li-ion diffusion coefficient was calculated from the plot between low-frequency Warburg region and Z’ (Ω) by employing Eq. 11^37^.

11 $${D}_{Li}^{+}=\frac{{R}^{2}{T}^{2}}{2{A}^{2}{n}^{4}{F}^{4}{c}^{2}{\sigma }^{2}}$$

where *R* is the gas constant, *T* is the absolute temperature, *A* is the area of electrode, *n* refers to the number of electrons involved in redox reaction, *F* is Faraday’s constant, *c* is the concentration of Li in active material, and *σ* is the Warburg coefficient obtained from the slope. The *D*_*Li*_^+^ values (Table S6) of P-LMO and LMO@C are 5.5 × 10^−15^, and 1.1 × 10^−14^, respectively. These results indicate that the carbon shell can also improve the Li ion conductivity. Note, the *D*_*Li*_^+^ values of LiMn_2_O_4_ material were widely reported in the value between the range of 10^−13^–10^−15^ cm^2^/s^86–88^. Moreover, after 1000 cycles of stability test at 1 C, the *D*_*Li*_^+^ of P-LMO cell is significantly decreased to 1.1 × 10^−15^ cm^2^/s, while LMO@C cell exhibits high *D*_*Li*_^+^ of 8.1 × 10^−15^ cm^2^/s. This evidence clearly shows the suppression of Mn dissolution over long cycling.

## Conclusions

In summary, the carbon nanoparticles were successfully coated on Mn-rich LiMn_2_O_4_ cathode forming the core-shell structure namely LMO@C by a scalable mechanofusion process, which is a solid-solid mixing process without using any solvent with a nearly 100% yield of production. The LMO@C exhibits superior rate-capability and cycling stability than P-LMO. The improved cycling stability can be mainly attributed to the suppression of Mn dissolution- migration-anode deposition (DMD) process which was investigated by the post-mortem analyses. Ex situ SEM/EDX, ICP-OES and ex situ XPS reveal the lower amount of Mn deposition on the anode surface of LMO@C with *ca*. 1.5–2 times lower than the P-LMO. The lower Mn dissolution results in a lesser extent of impedance growth and better Li^+^ diffusion coefficient. Moreover, both the P-LMO and LMO@C show the generation of λ-MnO_2_ mixed with Li_1-δ_Mn_2_O_4_ phases as it can be observed from the rapidly dropped in the lattice parameter when Li was deintercalated over *ca*. 70–80%. However, the LMO@C exhibits lower change in the lattice parameter compared with the P-LMO, indicating to the lower generation of λ-MnO_2_ phase. Overall, the LMO@C can lessen the DMD process as well as minimize the phase transition leading to an improved cycling stability in 18,650 cylindrical batteries.

## Methods

### Synthesis of LMO@C core@shell materials

The carbon-coated LiMn_2_O_4_ with the core-shell structure (LMO@C) was synthesized by the solvent-free mechanofusion process using NOBILTA machine (NOM-130, Hosokawa Micron Corporation, Japan). Firstly, 90 wt.% of LiMn_2_O_4_ was transferred to the mechanofusion chamber and operated at 1000 rpm for 20 min. Then, the 10 wt.% of conductive carbon (Super P, TIMCAL) was added without any solvent and operated at 5000 rpm for 30 min.

### Physical characterization

The morphologies of the P-LMO and the as-synthesized LMO@C were characterized by field-emission scanning electron microscopy (FESEM, JSM7001F, JEOL Ltd.) and transmission electron microscopy (TEM, JEM-ARM200F, JEOL Ltd.). The structure of materials was investigated by X-ray diffraction (XRD, Bruker, D8 Advance) using a Cu Kα radiation (λ = 1.54056 Å). The Rietveld refinement was calculated using the Raetia software. The lattice parameters of *in operando* XRD were calculated using the TOPAS software by excluding the Bragg reflections from Al and Be windows. The element composition was explored by X-ray photoelectron spectroscopy (XPS, Kratos Axis Ultra DLD) using the monochromatic Al-Kα (1486.7 eV) as the excitation source and C 1 s (284.6 eV) for a reference binding energy. The amount of Mn dissolution and deposition on the anode side was obtained from an inductively coupled plasma−optical emission spectrometry (ICPOES, Agilent Technologies 700 series). Raman spectra were collected from a dispersive Raman spectrometer (SENTERRA, Bruker) with an excitation wavelength of 532 nm. Thermogravimetric analysis (TGA) was performed using STA PT1600 under an oxygen atmosphere at a heating rate of 10 °C min^−1^. The electrical resistivity of electrodes was measured using a four-point probe technique (JANDLE, Model RM3000+, UK). For *in operando* XRD, the P-LMO and LMO@C specimens were prepared in the *in operando* XRD cell (LRCS, Amiens) coupled with the XRD machine (Bruker) and Autolab (PGSTAT 302 N). The XRD spectrum was collected every 7.8 min at 2 Theta of 3–80° during charging and discharging.

### Coin-cell fabrication and electrochemical evaluation

The P-LMO and LMO@C electrodes were fabricated by mixing of active materials, carbon black, and PVDF in a weight ratio of 8:1:1 dissolved in NMP and sonicated for 2 h. The as-prepared slurry was coated on the Al foil with a mass loading of *ca*. 5–6 mg per a current collector area of 1.96 cm^2^. The CR2032 coin cells were fabricated using the as-prepared cathode with Li foil anode assembled in an argon-filled glove box (Mbraun labstar glove box workstation). 1.0 M LiPF_6_ in EC: DEC (1: 1 by volume) was used as an electrolyte. The commercial polypropylene (Celgard) was used as a separator. The electrochemical performance of batteries was tested using Galvanostatic charge-discharge (GCD, NEWARE battery tester).

### Fabrication of cylindrical 18650 LIBs and electrochemical evaluation

All the fabrication processes were performed in a dry room with a dew point temperature of −40 °C except for the electrolyte injection process at −55 °C. For the cathode slurry preparation, the P-LMO and LMO@C were prepared by mixing of active materials, carbon black, and PVDF in a weight ratio of 0.956: 0.022: 0.022 in NMP and stirred for 12 h using a vacuum planetary mixer (MTI Corp.). On the other hand, the graphite anode slurry was prepared by mixing 0.966: 0.017: 0.017 weight ratio of graphite, carbon black, and CMC/SBR in water. Next, the as-prepared slurries were coated using a roll-to-roll automatic coating machine with a built-in dryer (MTI Corp.) at 120 °C. The thicknesses of cathode and anode were controlled at *ca*. 240 µm and 228 µm, respectively. The electrodes were processed through pressing, rolling, slitting, cutting, winding, electrolyte injection, and crimping. The EJN04 commercial LMO electrolyte (Gelon LIB) and a tri-layer PP/PE/PP were used as the electrolyte and the separator, respectively. For the capacity determination, the fabricated cells were charged to 4.2 V at 0.1 C (C = 1400 mA) using CCCV and discharged to 3.0 V at 0.1 C. The rate capability test was performed using CCCV at 0.1 C, 0.25 C, 0.5 C, 0.75 C, and 1 C. Cycling test was carried out at 1 C for 900 cycles. Electrochemical Impedance Spectroscopy (EIS) was conducted by using half-cell CR2032 with an electrochemical workstation (AUTOLAB, PGSTAT302N) over a frequency range of 0.01–100 kHz with an amplitude of 10 mV.

## Supplementary information

Supplementary Information

## Data Availability

The data generated during this study are included in the paper and the Supplementary Information.
