# Supplementary material for: Core-shell structure of LiMn2O4 cathode material reduces phase transition and Mn dissolution in Li-ion batteries
Source: Commun Chem. 2022 Apr 19;5:54. doi: 10.1038/s42004-022-00670-y (PMC9814138; doi:10.1038/s42004-022-00670-y)
Supplement: Supplementary file 1 — Supplementary Information [file 42004_2022_670_MOESM1_ESM.pdf]

Supplementary information

Core-shell structure of $LiMn_2O_4$ cathode material reduces phase transition and Mn dissolution in Li-ion batteries

Chanikarn Tomon,[a] Sangchai Sarawutanukul,[a] Nutthaphon Phattharasupakun,[a] Salatan Duangdangchote,[a] Praeploy Chomkhuntod,[a] Nattanon Joraleechanchai,[a] Panyawee Bunyanidhi and Montree Sawangphruk [a,*]

[a]Center of Excellence for Energy Storage Technology (CEST), Department of Chemical and Biomolecular Engineering, School of Energy Science and Engineering, Vidyasirimedhi Institute of Science and Technology, 555 Moo 1 Payupnai, Wangchan District, Rayong 21210, Thailand

*Corresponding author: montree.s@vistec.ac.th

Supporting Figures

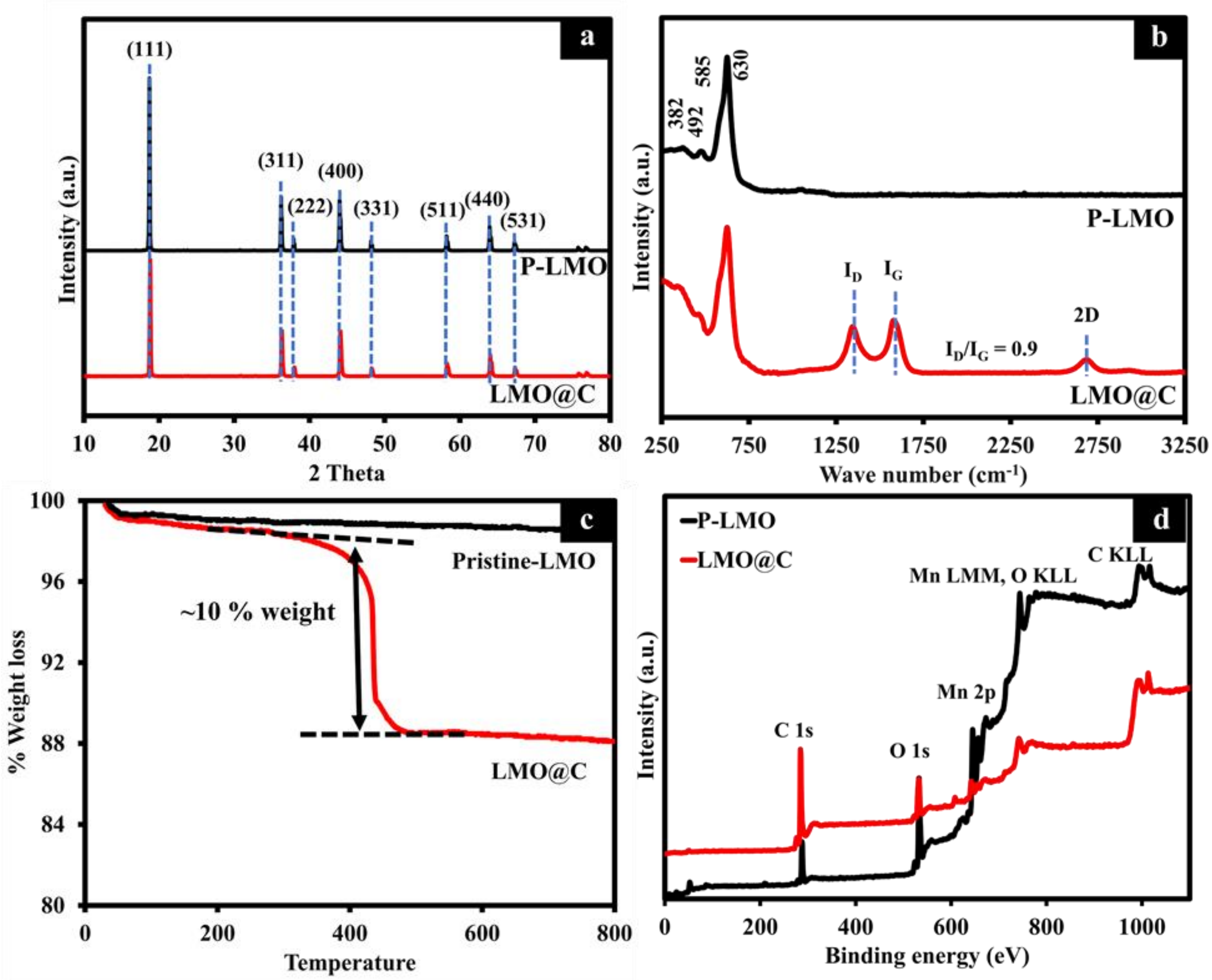

Figure S1. **Physical characterization of P-LMO and LMO@C.** **(a)** XRD patterns of P-LMO and LMO@C. **(b)** Raman spectra of P-LMO and LMO@C. **(c)** TGA results of P-LMO and LMO@C. **(d)** Wide-scan XPS spectra of P-LMO and LMO@C.

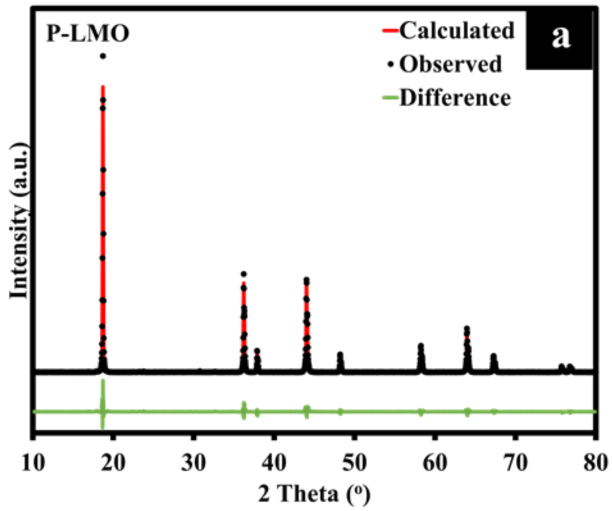

Figure S2. **XRD Rietveld refinements of P-LMO and LMO@C.** (a-b) Refinement results of (a) P-LMO, and **(b)** LMO@C.

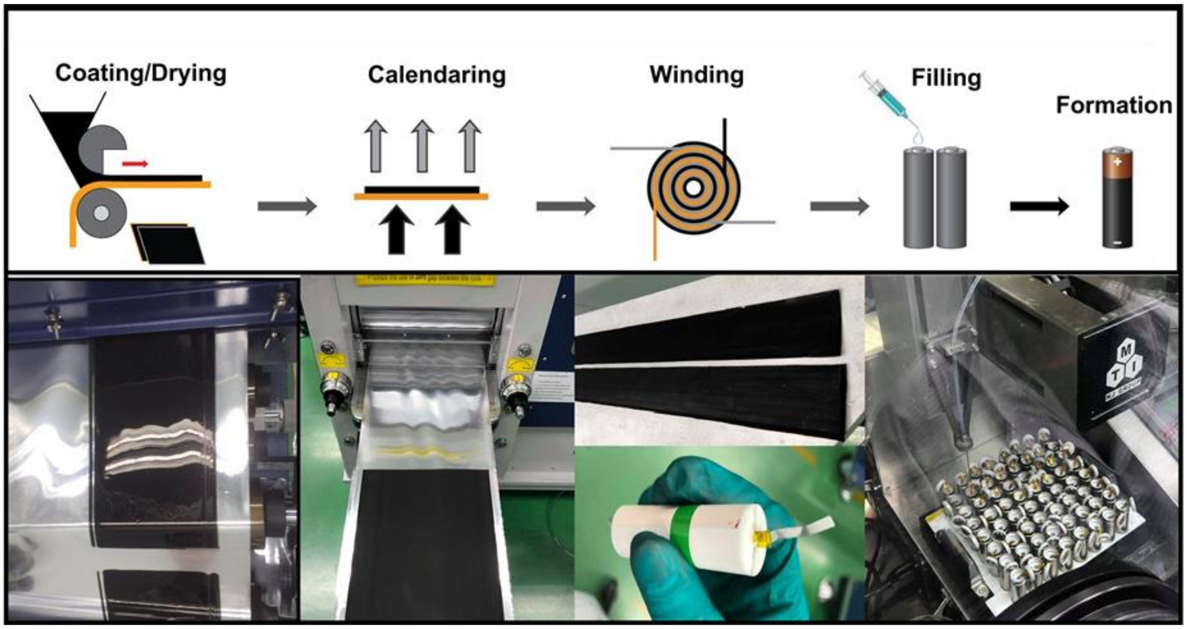

Figure S3. Schematic illustrations and photographs of the 18650 LIBs manufacturing process in a dry room with a dew point temperature of -40 °C and especially -55 °C for the electrolyte injection unit.

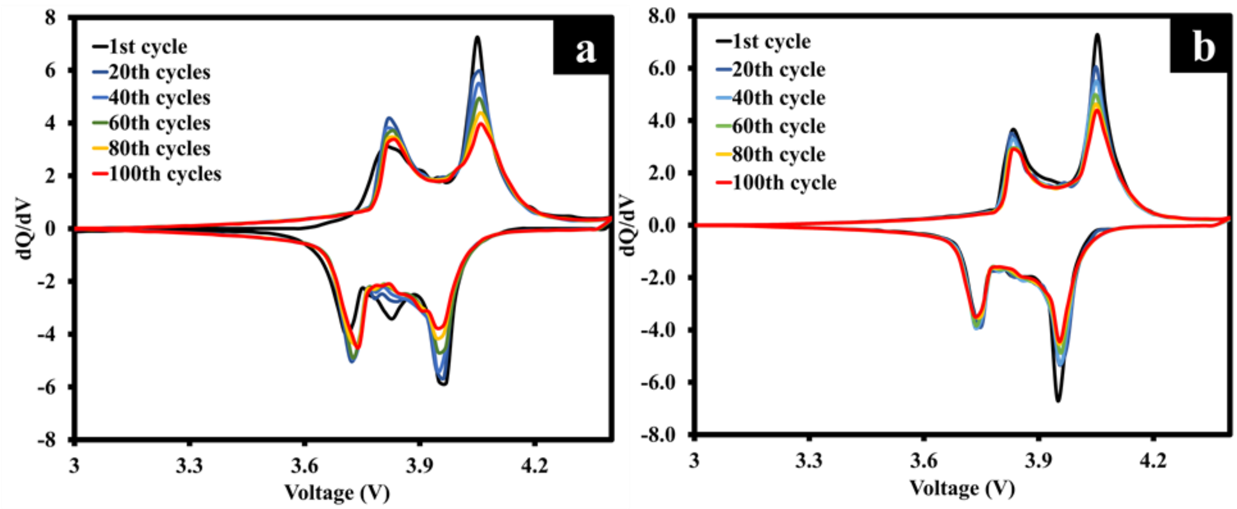

Figure S4. The dQ/dV represented Li^+ deintercalation/intercalation over cycling. (a-b) The dQ/dV curves of **(a)** P-LMO and **(b)** LMO@C.

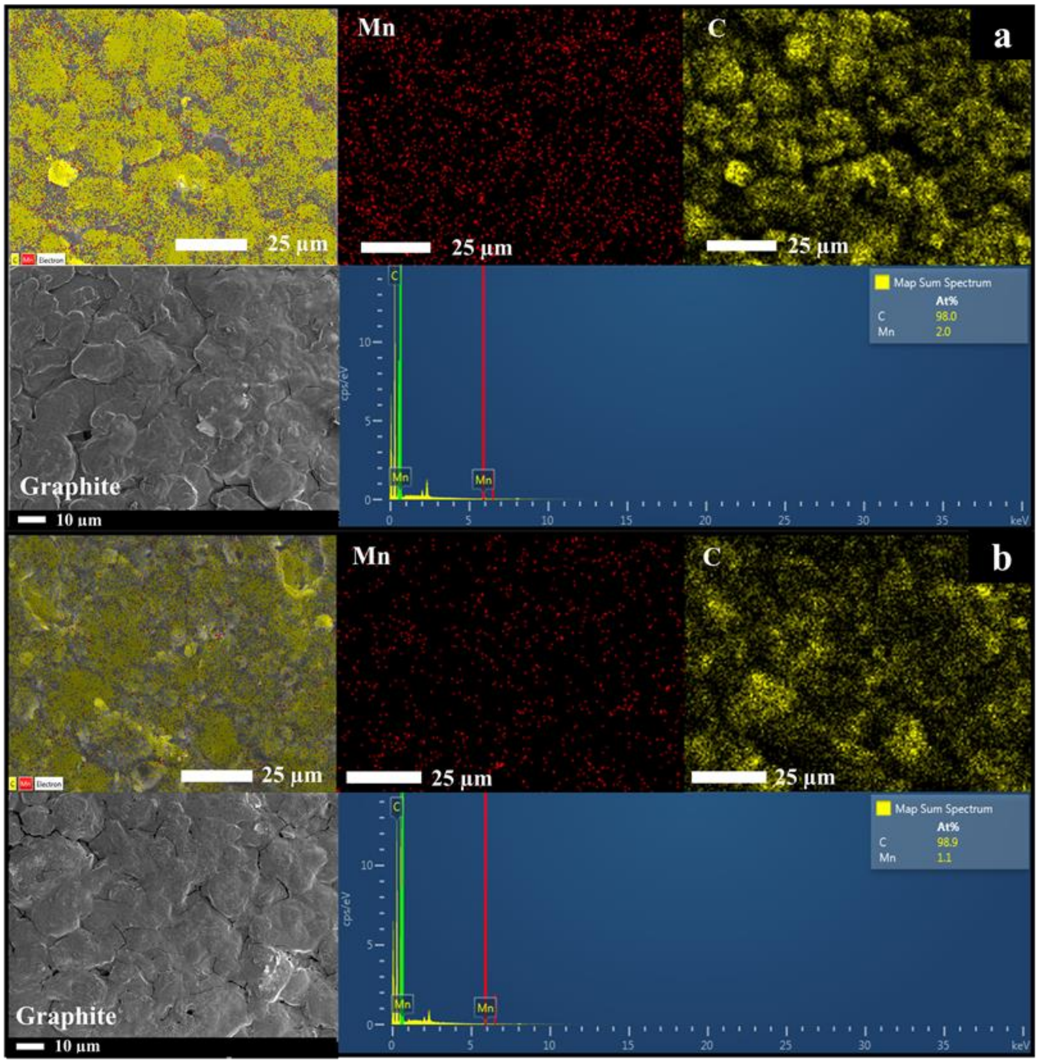

Figure S5. ***Ex situ* SEM images and EDS mapping of graphite anode after cycling. (a-b)** SEM and EDS of graphite electrodes **(a)** disassembled from the P-LMO//graphite, and **(b)** LMO@C//graphite after 200 cycles.

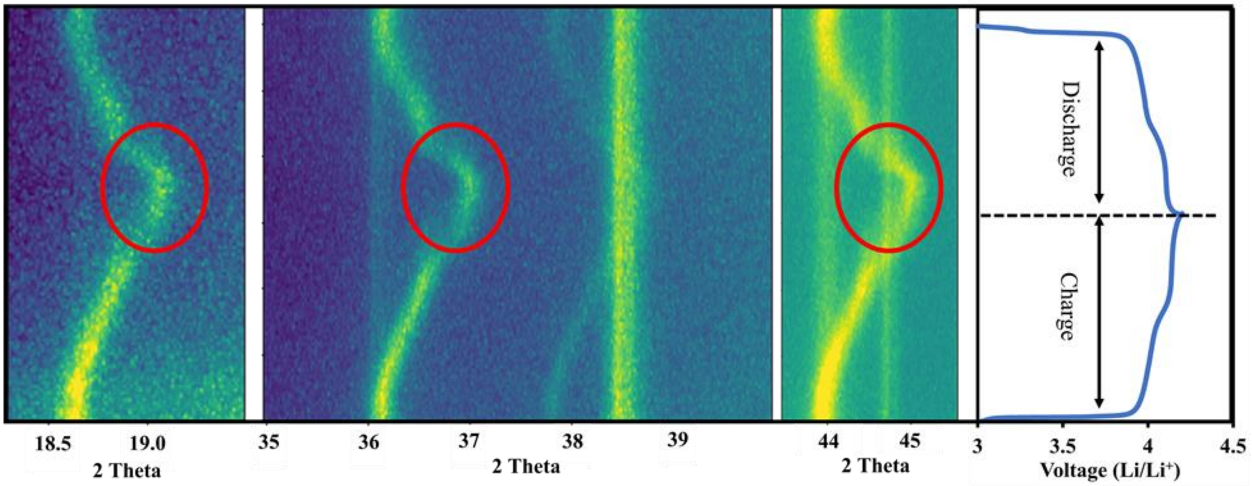

Figure S6. ***In operando* XRD of P-LMO//graphite during charge and discharge processes**.

Supporting Tables

Table S1. Rietveld refinement data of P-LMO and LMO@C.

	P-LMO	LMO@C
Lattice parameter (Å)	a=b=c= 8.23 (± 7.13 x 10^{-5})	a=b=c= 8.23 (± 2.15 x 10^{-4})
V ($Å^3$)	557.91	558.15
Bragg R-factor	4.83	4.79

Table S2. The results from four-point probe measurement.

<table>
<tr><td></td><td colspan="2">LMO</td><td colspan="2">LMO@C</td><td colspan="2">Graphite</td></tr>
<tr><td rowspan="2">Thickness</td><td colspan="2">230 μm</td><td colspan="2">228 μm</td><td colspan="2">228 μm</td></tr>
<tr><td>Forward scan</td><td>Reverse scan</td><td>Forward scan</td><td>Reverse scan</td><td>Forward scan</td><td>Reverse scan</td></tr>
</table>

Applied current at 100 μA	2.36 mΩ/cm	2.14 mΩ/cm	0.473 mΩ/cm	0.471 mΩ/cm	1.30 mΩ/cm	1.03 mΩ/cm

Table S3. The ICP-OES results of P-LMO and LMO@C after cycling.

**Sample**	**Element**	**MW (mg/mol)**	**R^2**	**Concentration found (mg/l)**
P-LMO	Mn	54938.04	0.999	0.02221
LMO@C	Mn	54938.04	0.999	0.0100

Table S4. *Ex situ* XPS results.

	C 1s	Mn 2p1/2	Mn/C
P_LMO	80.20985	19.79015	0.24673
LMOCS	85.47935	14.52065	0.169873

Table S5. The R_s and R_{ct} values of P-LMO and LMO@C before and after cycling.

Samples	R_s	R_{ct}	CPE	N	Y_2	X^2
			Y_1			
P-LMO 1	1.5	144	20.7 x 10^{-6}	0.79	2.82 x 10^{-3}	0.081
P-LMO 1000	1.64	230	7.9 x 10^{-6}	0.85	6.82 x 10^{-3}	0.086
LMO@C 1	2.34	78	23.1 x 10^{-6}	0.81	1.43 x 10^{-3}	0.082
LMO@C 1000	1.8	97.6	15.4 x 10^{-6}	0.78	8.43 x 10^{-3}	0.084

Table S6. The Li-ion diffusion coefficient (D_{Li}^+) of P-LMO and LMO@C.

Samples	D_{Li}^+
LMO@C 1st cycle	1.1 x 10^{-14}

LMO@C after 1000 cycles	8.1 x 10^{-15}
P-LMO 1st cycle	5.5 x 10^{-15}
P-LMO after 1000 cycles	1.1 x 10^{-15}
